# Case Report: Recurrent abdominal pain with elevated pancreatic enzymes in type 2 diabetes mellitus: a case of IgG4-related autoimmune pancreatitis misdiagnosed as acute pancreatitis

**DOI:** 10.3389/fendo.2026.1893666

**Published:** 2026-07-13

**Authors:** Yaping Shi, Wanying Xue, Zhe Dai, Yuwen Wu

**Affiliations:** 1 Department of Endocrinology, Zhongnan Hospital of Wuhan University, Wuhan, China; 2 Department of Clinical Nutrition, Zhongnan Hospital of Wuhan University, Wuhan, China; 3 State Key Laboratory of Metabolism and Regulation in Complex Organisms, College of Life Sciences, Wuhan University, Wuhan, China

**Keywords:** acute pancreatitis, glucocorticoid, IgG4-related autoimmune pancreatitis, misdiagnosis, type 2 diabetes mellitus

## Abstract

**Background:**

IgG4-related autoimmune pancreatitis (IgG4-AIP) is a rare autoimmune pancreatic disorder with non-specific clinical manifestations that is often misdiagnosed as acute pancreatitis (AP). Its coexistence with type 2 diabetes mellitus (T2DM) may further complicate recognition of the underlying pancreatic disease.

**Case presentation:**

A 60-year-old male with a 4-year history of T2DM was admitted for diabetic complications manifesting as blurred vision and lower limb numbness. During hospitalization, he developed recurrent epigastric pain associated with elevated pancreatic enzymes and was initially diagnosed with AP. Conventional treatment for AP did not lead to sustained improvement, and amylase and lipase remained persistently elevated. Serum IgG4 was markedly elevated at 13.8 g/L. Imaging findings were unremarkable. Endoscopic ultrasound-guided fine-needle aspiration revealed chronic inflammatory changes. The diagnosis of IgG4-AIP was ultimately established based on the markedly elevated serum IgG4 level, supportive (though atypical) imaging findings, histopathological features, and a favorable response to glucocorticoid therapy. The patient’s symptoms improved, accompanied by an overall decrease in serum IgG4 and pancreatic enzyme levels.

**Conclusion:**

In T2DM patients with recurrent abdominal pain and persistently elevated pancreatic enzymes unresponsive to routine AP treatment, IgG4-AIP should be considered. Timely measurement of serum IgG4 and careful integration of imaging, histopathology, and treatment response are important for early diagnosis. Early recognition may prevent misdiagnosis and enable timely initiation of glucocorticoid therapy.

## Introduction

IgG4-related autoimmune pancreatitis (IgG4-AIP), a pancreatic manifestation of IgG4-related disease, is a rare chronic inflammatory disorder that predominantly affects older males (1). IgG4-AIP is typically characterized by lymphoplasmacytic infiltration, fibrosis, elevated serum IgG4 in many patients, and variable pancreatic imaging findings. Its clinical manifestations are non-specific; while painless jaundice is the primary clinical symptom, patients occasionally present with abdominal pain and elevated pancreatic enzymes that mimic acute pancreatitis (AP) (2, 3). IgG4-AIP is easily misdiagnosed and delayed due to its low incidence and overlapping clinical features with more common pancreatic diseases. Moreover, endocrine dysfunction and coexisting type 2 diabetes mellitus (T2DM) are common features in IgG4-AIP, where pancreatic inflammation may impair islet function and exacerbate glycemic abnormalities (4, 5). Here, we report a case of a 60-year-old male with T2DM who developed recurrent abdominal pain and elevated pancreatic enzymes during hospitalization, posing a considerable diagnostic challenge.

## Case presentation

### Chief complaints

A 60-year-old male was admitted to our hospital due to a 10-day history of blurred vision and lower limb numbness.

### Medical history

The patient had a 4-year history of T2DM with suboptimal glycemic control despite treatment with oral hypoglycemic agents and insulin. He also had a history of hypertension, gout, and an episode of pancreatitis 4 months prior to the current admission. There was no history of surgery, trauma, or known drug allergies.

### Physical examinations

On admission, his temperature was 36.7°C, blood pressure was 114/77 mmHg, pulse rate was 74 beats/min, and body mass index was 24.86 kg/m². He was alert and hemodynamically stable. No jaundice, lymphadenopathy, or lower-extremity edema was observed. Cardiopulmonary examination was unremarkable; the abdomen was soft without tenderness.

### Laboratory examinations

On admission, laboratory examinations showed poor glycemic control with a fasting blood glucose of 14.01 mmol/L (reference, 3.9–6.1 mmol/L), a glycated hemoglobin of 7.9% (4.0%–6.0%) and an elevated blood ketone level of 0.91 mmol/L (reference, 0–0.28 mmol/L). Renal and metabolic abnormalities included elevated blood urea nitrogen at 9.81 mmol/L (2.8–7.6 mmol/L), serum creatinine at 112.8 μmol/L (64–104 μmol/L), uric acid at 479.5 μmol/L (208–428 μmol/L), triglycerides at 2.46 mmol/L (<1.70 mmol/L), and cystatin C at 1.30 mg/L (0.59–1.03 mg/L). Urinalysis showed glucose 4^+^, occult blood 1^+^, and leukocyte esterase 2+ (reference, normally negative). Serum amylase and lipase were 100 U/L and 81 U/L (reference range, amylase: 0–90 U/L; lipase: 0–70 U/L), respectively. Following the onset of acute abdominal pain on the fifth day of admission, repeat testing showed recurrent elevations of pancreatic enzymes. Further etiologic evaluation revealed a markedly elevated serum IgG4 level of 13.8 g/L, whereas the antinuclear antibody and extractable nuclear antigen panel were negative. During hospitalization and subsequent outpatient follow-up, repeated assessments of gastrointestinal tumor markers, including CEA, CA19-9, CA72-4, and AFP, remained within normal ranges. An oral glucose tolerance test showed persistently elevated glucose levels, with a preserved but delayed C-peptide release, suggesting impaired islet function.

### Imaging examinations

Initial pancreatic imaging of ultrasound and CT showed no typical features of AP. Following the onset of abdominal pain, emergency abdominal CT again revealed a lack of typical imaging findings associated with AP, although mild parenchymal fullness of the pancreatic body and tail was noted (**Figure 1A**). Subsequent contrast-enhanced CT and MRI/MRCP confirmed the fullness of the pancreatic body and tail, with no evidence of a discrete pancreatic mass, significant pancreatic duct dilatation, or distinct signal intensity abnormalities, thereby rendering pancreatic malignancy unlikely (**Figure 1B**). No new pancreatic mass or progressive ductal abnormality has been identified during follow-up imaging evaluations to date. Endoscopic ultrasound-guided fine-needle aspiration showed chronic inflammatory changes consistent with chronic pancreatitis, with no evidence of malignant cells in the sampled specimen, although immunohistochemical staining for IgG4 was non-specific (**Figure 2**).

**Figure 1:**
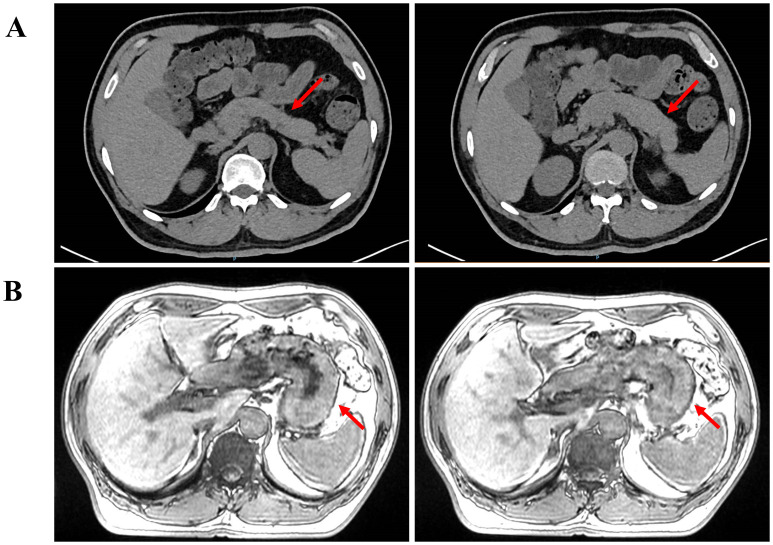
Pancreatic imaging findings. **(A)** Abdominal CT revealing mild parenchymal fullness of the pancreatic body and tail (arrows), with no typical imaging features of acute pancreatitis. **(B)** MRI showing mild fullness of the pancreatic body and tail (arrows), without distinct signal intensity abnormalities. CT, computed tomography; MRI, magnetic resonance imaging.

**Figure 2:**
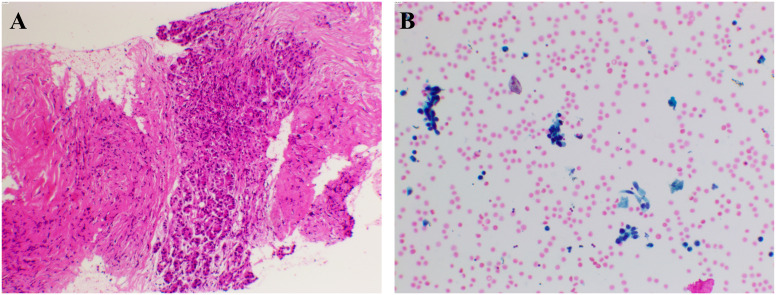
Histopathological findings of EUS-FNA of the pancreas. **(A)** H&E staining (×200) of the core biopsy specimen showing prominent inflammatory cell infiltration predominantly composed of neutrophils within a fibrotic stroma, interspersed with a small volume of preserved, well-differentiated pancreatic acinar cells and occasional plasma cells, suggestive of chronic inflammatory changes. **(B)** Liquid-based cytology with Papanicolaou staining (×200) revealing a small number of well-differentiated pancreatic acinar cells and ductal epithelial cells, with no evidence of atypical epithelial cells. EUS-FNA, endoscopic ultrasound-guided fine-needle aspiration; H&E, hematoxylin & eosin.

### Final diagnosis

Following a gastroenterology consultation, an initial working diagnosis of AP was considered because of the acute abdominal pain and elevated pancreatic enzyme levels. However, subsequent reassessment showed that the overall clinical and imaging findings were not fully consistent with typical acute pancreatitis. Given the persistent abdominal pain, fluctuating pancreatic enzyme levels, atypical imaging findings, and the lack of sustained response to conventional anti-pancreatitis therapy, the case was reassessed. Considering the markedly elevated serum IgG4 level (>2 times the upper limit of normal), multimodal imaging showing pancreatic inflammatory changes without pancreatic malignancy or other space-occupying lesion, histopathologic evidence of chronic pancreatic inflammation, and the marked decline in serum IgG4 and pancreatic enzyme levels to glucocorticoid therapy, the overall clinical picture was considered most consistent with IgG4-AIP. The overall clinical findings were most consistent with type 1 IgG4-related autoimmune pancreatitis according to the International Consensus Diagnostic Criteria (ICDC, 2011), based on an integrated assessment of serology, imaging findings, exclusion of alternative diagnoses, and response to glucocorticoid therapy, alongside coexisting T2DM and multiple chronic comorbidities.

### Treatment

Following an initial course of conventional treatment for presumed AP, including fasting, analgesia, acid suppression, octreotide, anti-infective therapy, and supportive care, the patient’s symptoms and pancreatic enzyme abnormalities did not show sustained improvement. After the diagnosis was revised to IgG4-AIP, prednisone was initiated at 40 mg/day, increased to 50 mg/day after one week based on changes in serum IgG4 levels and clinical manifestations, and subsequently tapered sequentially to 40 mg/day and 30 mg/day before being gradually reduced during follow-up to a maintenance dose of 7.5 mg/day. The antidiabetic regimen was adjusted accordingly. Supportive treatment for his other comorbid conditions was maintained.

### Outcome and follow-up

Following the initiation of glucocorticoid therapy, the patient’s abdominal pain resolved, accompanied by a reduction in serum IgG4 and pancreatic enzyme levels. On follow-up, serum IgG4 decreased from 13.8 g/L to 3.98 g/L, while amylase and lipase normalized to 86 U/L and 45 U/L, respectively (**Figure 3**). Blood glucose levels and other metabolic indices gradually stabilized after adjustment of his treatment regimen. Although interim laboratory fluctuations were observed, the overall clinical and biochemical improvement after prednisone therapy supported the diagnosis of IgG4-AIP. The patient remains under regular clinical and imaging follow-up, with no evidence of pancreatic malignancy detected to date.

**Figure 3:**
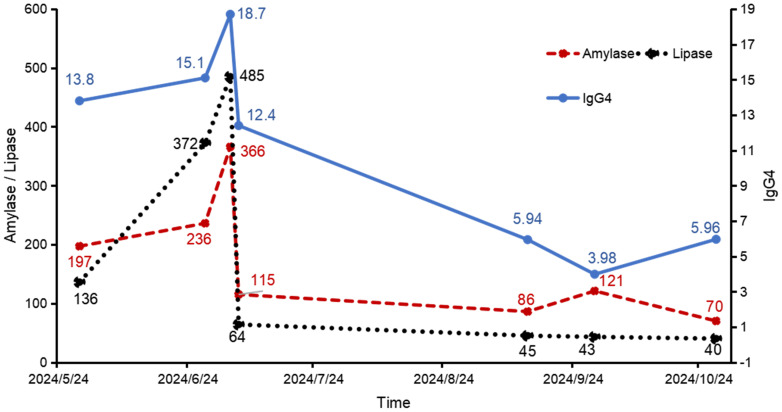
Dynamic trends of serum IgG4, amylase, and lipase throughout the clinical course. All three parameters showed an overall decline following the initiation of glucocorticoid therapy, notwithstanding interim fluctuations.

## Discussion

The present case illustrates a highly atypical clinical presentation of IgG4-AIP. A 60-year-old male patient with T2DM developed recurrent epigastric pain and fluctuating elevations of amylase and lipase during hospitalization, leading to an initial working diagnosis of AP. This atypical presentation, together with the coexistence of diabetes and multiple metabolic comorbidities, contributed to the initial diagnostic confusion.

The diagnostic challenge in this case arose from the overlap between IgG4-AIP and more common pancreatic disorders. According to the ICDC, the diagnosis of AIP relies on a combination of pancreatic imaging, serology, other organ involvement, pancreatic histopathology, and response to steroid therapy (6). Under the ICDC framework, histopathological confirmation is not mandatory when the required combination of diagnostic criteria is fulfilled. Based on these criteria, the present case was considered to fulfill the diagnostic category of definitive type 1 AIP. In clinical practice, however, these diagnostic features are not always simultaneously typical. In our patient, the initial CT and subsequent MRI/MRCP did not show the classic radiologic features of AIP, such as diffuse “sausage-like” enlargement or a characteristic pancreatic duct abnormality (2, 3). Instead, imaging showed only parenchymal fullness of the pancreatic body and tail without definitive signal abnormalities, which was insufficient on its own to establish the diagnosis (3, 7). Of clinical importance, unremarkable imaging findings do not exclude IgG4-AIP when the overall clinical picture remains suspicious. Although acute pancreatitis and IgG4-AIP are not mutually exclusive and concomitant acute pancreatitis cannot be completely excluded, repeated CT examinations in this patient failed to demonstrate typical imaging features of acute pancreatitis. Moreover, the persistent pancreatic enzyme abnormalities despite conventional anti-pancreatitis therapy and the marked improvement after glucocorticoid treatment suggested that IgG4-AIP was the underlying disease process accounting for the patient’s overall clinical course.

Serology provided the most important diagnostic indicator in this patient. Serum IgG4 is a useful diagnostic marker for type 1 AIP, although it is not entirely disease-specific (2). Mild to moderate elevations may also occur in pancreatic cancer, chronic pancreatitis, and other inflammatory or autoimmune conditions (8). Nevertheless, the serum IgG4 level in this case was markedly elevated to 13.8 g/L, far above the commonly used diagnostic threshold (0.03–2.01 g/L), therefore substantially increasing the likelihood of IgG4-AIP in the appropriate clinical context (6). Importantly, the interpretation of serum IgG4 should always be integrated with imaging, histopathology, and clinical evolution rather than used in isolation.

Histopathology in this case must be interpreted cautiously. Classic histologic features of IgG4-AIP include lymphoplasmacytic infiltration, storiform fibrosis, and abundant IgG4-positive plasma cells (9). In our patient, endoscopic ultrasound-guided fine-needle aspiration (EUS-FNA) primarily demonstrated chronic inflammatory changes suggestive of chronic pancreatitis, while immunohistochemical staining for IgG4 was reported as non-specific rather than definitively diagnostic. Therefore, the diagnosis in this case was established based on an integrated assessment of the ICDC criteria, including markedly elevated serum IgG4, compatible imaging findings, histopathologic features, exclusion of alternative diagnoses, and a favorable response to glucocorticoid therapy.

In addition, the atypical imaging findings and non-specific IgG4 staining in this case may be related to the stage of the disease. It is possible that the patient was in an early phase of IgG4-AIP, a period in which characteristic radiologic features and typical histopathological findings may not yet be fully developed (2, 3). With disease progression, more typical imaging changes and increased IgG4-positive plasma cell infiltration often become more evident. Furthermore, the limited tissue volume obtained via EUS-FNA may have contributed to the non-specific histopathological findings. Compared with surgical biopsy, EUS-FNA samples are smaller and may not adequately capture the characteristic features of IgG4-AIP, potentially leading to the underestimation of IgG4-positive cell infiltration (9). Although type 2 AIP should theoretically be included in the differential diagnosis, the markedly elevated serum IgG4 level (>2 times the upper limit of normal) strongly supported IgG4-AIP in this patient. Moreover, granulocytic epithelial lesions, a characteristic histopathologic feature of type 2 AIP, were not observed in the present case (10).

Another notable feature of this case is the coexistence of T2DM. Endocrine dysfunction is common in AIP, and pancreatic inflammation may contribute to impaired glucose metabolism, likely through its impact on islet cell function (4, 11). Conversely, in a patient already known to have diabetes, new abdominal symptoms and pancreatic enzyme abnormalities may be too easily attributed to metabolic stress or AP, delaying the consideration of autoimmune pancreatic disease (3). In the present case, the background of T2DM and its complications likely diverted early attention toward more common metabolic and gastrointestinal explanations. This suggests that in diabetic patients with recurrent abdominal pain, fluctuating pancreatic enzymes, and a transient response to conventional AP treatment, clinicians should broaden the differential diagnosis and consider IgG4-AIP early in the evaluation.

The treatment response in this patient further validated the diagnosis. Glucocorticoids remain the first-line therapy for IgG4-AIP, and prednisone at an initial dose of 0.6–0.8 mg/kg/day is commonly recommended, followed by tapering according to the clinical and biochemical response (12). Our patient received prednisone 40–50 mg daily, based on changes in serum IgG4 levels and clinical manifestations, resulting in the resolution of abdominal pain and an overall decrease in serum IgG4 and pancreatic enzyme levels, notwithstanding minor interim fluctuations in laboratory values. This pattern is clinically meaningful, in IgG4-AIP, improvement after steroid therapy is both therapeutically important and diagnostically informative when interpreted alongside the other available evidence (2). For refractory, relapsing, or steroid-dependent disease, immunosuppressive agents or rituximab may be considered (13).

This case offers several clinical implications. First, IgG4-AIP should be considered in patients with recurrent or unexplained pancreatic enzyme elevations, especially when the clinical course is inconsistent with typical AP or the response to conventional therapy is unsatisfactory. Second, the absence of classic radiologic findings does not exclude the diagnosis; rather, atypical cases require close integration of serology, tissue findings, and treatment response. Third, histopathology obtained by minimally invasive sampling may be supportive but is not always definitive, and clinicians should avoid overstating the diagnostic certainty of limited biopsy specimens. Finally, the management of IgG4-AIP in patients with T2DM requires attention to both immunologic control and metabolic safety, because glucocorticoid therapy may alter glycemic management and necessitates adjustment of antidiabetic treatment.

In summary, this case highlights an atypical and insidious presentation of IgG4-AIP mimicking AP in a patient with T2DM. Notably, the presence of diabetic ketosis during episodes of abdominal pain may further obscure the clinical picture and contribute to diagnostic difficulty. The final diagnosis relied upon a stepwise clinicopathologic assessment rather than a single definitive finding. Increased awareness of this presentation is essential to mitigate misdiagnosis and diagnostic delays, particularly in diabetic patients presenting with recurrent abdominal pain, fluctuating pancreatic enzymes, and inconclusive imaging findings.

## Data Availability

The original contributions presented in the study are included in the article/supplementary material. Further inquiries can be directed to the corresponding authors.
